# Skin mucormycosis presenting as an erythema-nodosum-like rash in a renal transplant recipient: a case report

**DOI:** 10.1186/1752-1947-2-112

**Published:** 2008-04-19

**Authors:** Nader Nouri-Majalan, Mansour Moghimi

**Affiliations:** 1 Nephrology Department, Sadoughi Medical University, Yazd, Iran; 2 Pathology Department, Sadoughi Medical University, Yazd, Iran

## Abstract

**Introduction:**

Cutaneous mucormycosis is a rare entity related to kidney transplantation. It usually presents with ecthyma-like lesions and black necrotic cellulitis. We report an unusual case of primary cutaneous mucormycosis presenting as erythema-nodosum-like lesions in a woman who had received a renal transplant.

**Case presentation:**

A 49-year-old woman with diabetes received a living-unrelated kidney transplant. Her clinical course was uneventful for the first six months after transplantation. She then developed multiple, painful, erythema-nodosum-like lesions on her right leg and thigh following an episode of minor trauma. Mucormycosis was diagnosed by skin biopsy. Microscopic examination also showed panniculitis. The patient was treated successfully with amphotericin B and surgical resection. To our knowledge, this is the first description of primary cutaneous mucormycosis with erythema-nodosum-like lesions and panniculitis after renal transplantation.

**Conclusion:**

Cutaneous mucormycosis should be considered in the differential diagnosis when a kidney transplant recipient develops erythema-nodosum-like lesions with panniculitis.

## Introduction

Mucormycosis is a rare but potentially lethal fungal infection that can develop in renal allograft recipients. Although the infection may be rhinocerebral, pulmonary, gastrointestinal or disseminated, the most common form in renal transplant recipients is rhinocerebral, whereas cutaneous involvement is rare [1]. Diagnosis is usually made only after histopathology or culture results are available. We describe here a renal transplant recipient with diabetes mellitus who, after a minor traumatic wound to the leg, developed an erythema-nodosum-like lesion which was diagnosed as mucormycosis.

## Case presentation

**Figure 1:**
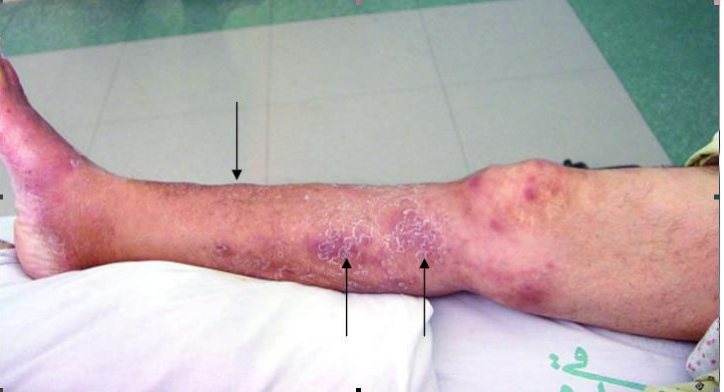
Erythema-nodosum-like lesions of the leg and thigh.

A 49-year-old woman who had developed end-stage renal disease as a result of diabetes and had been on maintenance CAPD (continuous ambulatory peritoneal dialysis) for 22 months underwent live-unrelated kidney transplantation in April 2006. Her clinical course was uneventful for the first six months after transplantation and she did not have any rejection episodes. She was maintained on cyclosporine (4 mg/kg/day), mycophenolate mofetil (2 g/day) and prednisolone (5 mg/day). Five months after transplantation, she presented with cellulitis of the right leg following minor trauma and was treated with intravenous cefazolin (4 g/day) and ceftriaxone (2 g/day). The signs and symptoms of cellulitis improved but 3 weeks later, multiple painful erythematous firm nodules of diameter 2 to 7 cm appeared on the anterior and posterior aspects of her right leg and the lower part of her right thigh, and some of the nodules became ulcerated (Figure 1) [Additional file 1]. The patient was febrile (39°C), her blood pressure was 130/90 mmHg, and her pulse rate was regular at 80/min. There was no evidence of edema or lymphadenopathy, and her systemic examination was unremarkable.

**Figure 2:**
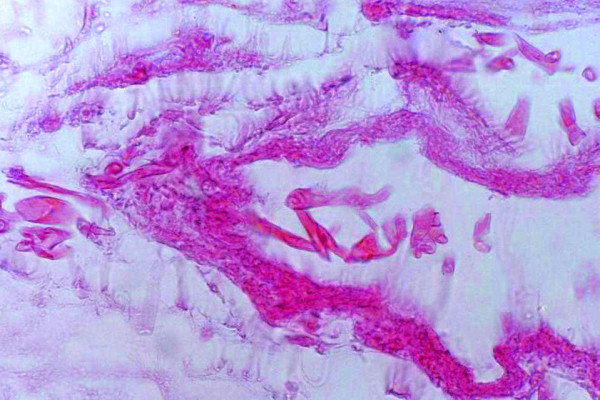
Histological section of the skin biopsy showing mucor hyphae in the vessel wall. and necrotic tissue (H&E × 400).

**Figure 3:**
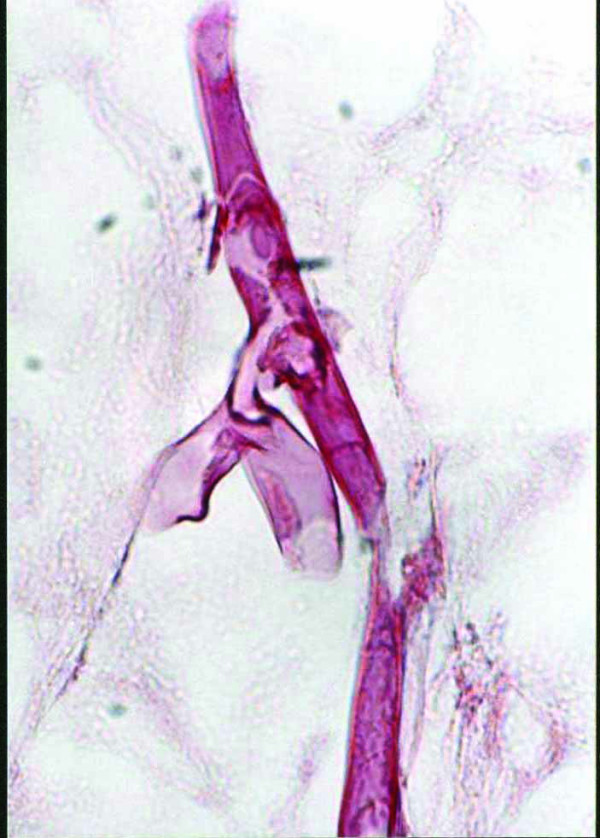
Broad, aseptate and thin walled fungal hyphae having irregular, non-parallel contours, with right angle branching indicative of mucormycosis (PAS × 1000).

Laboratory tests showed her hemoglobin was 8.6 g/dL, her total white cell count was 100,000/mm^3^, and her ESR was 125 mm for the first hour. Serum biochemistry showed her creatinine was 1.1 mg/dL, her fasting blood sugar was 191 mg/dL, her uric acid was 2.6 mg/dL and her lactate dehydrogenase was 625 IU/L. She was negative for cytomegalovirus (CMV) IgM, but positive for CMV IgG, which had also been positive prior to pretransplantation. Her chest X-ray was unremarkable. Right leg MRI showed severe thickening of the superficial soft tissue and skin, but no evidence of deep soft tissue or muscular structure involvement. Histological examination of deep incisional biopsies of some of the nodules revealed lobular panniculitis with infiltrating lymphocytes, neutrophils, multinucleated giant cells, foamy macrophages, fat necrosis and granulation tissue [Additional file 2]. Hematoxylin-eosin (H&E) and periodic acid schiff (PAS) staining showed numerous broad, aseptate and irregularly branched fungal hyphae indicative of mucormycosis deposited within the hypoderm and vessel wall (Figure 2) (Figure 3) [Additional file 3]. Specimen culture was negative for zygomycetes.

Since a fungal etiology of the skin lesion was established, the involved tissues were surgically resected, followed by intravenous treatment with amphotericin B (1 mg/kg/day). After 7 days of antifungal therapy, the patient's serum creatinine concentration had increased to 2.5 mg/dL; hence we had to decrease the dose of amphotericin B to 0.5 mg/kg/daily, and continue treatment until she had received a total cumulative dose of 1200 mg amphotericin B over 6 weeks. Mycophenolate mofetil was discontinued during anti-fungal therapy. The skin lesions showed a dramatic response to therapy, with only hyperpigmentation remaining in the involved areas of the skin. Following cessation of amphotericin B, her serum creatinine concentration decreased to 1.1 mg/dL and remained stable.

## Discussion

Zygomycetes are ubiquitous fungi belonging to the order Mucorales and the genera Rhizopus, Absidia, and Mucor [2] These fungi can cause a variety of infections in humans, including rhinocerebral [1], pulmonary [3], gastrointestinal [4], cutaneous and allograft [5] mucormycosis.

Risk factors predisposing to this disease include diabetes mellitus, solid organ transplantation, hematologic malignances and trauma and burns. Organ transplant recipients with concomitant diabetes mellitus are most susceptible to developing this infection [6]. The immunosuppressive effect of concomitant CMV infection, along with an increased risk of superinfection with opportunistic pathogens, is well established in transplant recipients [7]. Although our patient had the two most important risk factors, being an organ transplant recipient with concomitant diabetes mellitus, she did not have CMV infection preceding the onset of fungal disease.

Primary cutaneous mucormycosis is a relatively rare entity in renal transplant patients. For example, only 19 of 310 (6.1%) recipients of live-related renal transplants had documented systemic fungal infections, with only 2 having mucormycosis; both of these had the rhinocerebral disease form, whereas no patient had cutaneous mucormycosis [8].

Infection of skin and soft tissues with zygomycetes results from inoculation of the spores into the dermis. Fungal entry into the dermis has been associated with intravenous catheters [9], insulin injection sites [10], laparotomy wounds or prior surgical drain sites [6] and trauma [11]. Our patient only had an epsidode of minor trauma to her leg.

Past reports of cutaneous mucormycosis describe patients presenting with ecthyma-like lesions and black necrotic cellulitis [9,10,12-14]. To our knowledge, this is the first case report of erythema-nodosum-like lesions presenting as cutaneous manifestations of mucormycosis.

In agreement with previous findings [3], mucormycosis in our patient was diagnosed only through the detection of typical fungal hypha in the infected tissue, whereas fungal culture was negative. The lack of regular septations may contribute to the difficulties in culturing zygomycetes from clinical specimens.

To our knowledge, our case is the first description of cutaneous mucormycosis-associated panniculitis after organ transplantation. Previous cases of infection-induced panniculitis have been found due to gram positive or gram negative bacteria, atypical mycobacteria, nocardia, candida and fusarium species [15].

We found that treatment with amphotericin B and surgical debridement led to a favorable outcome. Similarly, previous patients with cutaneous mucormycosis and no evidence of hematogenous dissemination have been reported to have recovered fully after extensive local debridement and treatment with amphotericin B [10,11].

## Conclusion

In conclusion, cutaneous mucormycosis should be considered in the differential diagnosis when a kidney transplant recipient develops erythema-nodosum-like lesions with panniculitis.

## Competing interests

The authors declare that they have no competing interests.

## Authors' contributions

NN was primarily responsible for the diagnosis and management of the patient, drafting of the manuscript, literature search, and submission and revision of the manuscript. MM was responsible for writing up the pathology report, providing the slides and editing the manuscript. All authors have read and approved the final manuscript.

## Consent

Written informed consent was obtained from the patient for publication of this case report and any accompanying images. A copy of the written consent is available for review by the Editor-in-Chief of this journal.

## Supplementary Material

Additional file 1 Erythema-nodosum-like lesions of the leg and thigh Click here for file

Additional file 2 Panniculitis Click here for file

Additional file 3 Broad, aseptate and thin walled fungal hyphae with irregular, non-parallel contours Click here for file
