# Supplementary figures and images for: Skin mucormycosis presenting as an erythema-nodosum-like rash in a renal transplant recipient: a case report
Source: J Med Case Rep. 2008 Apr 19;2:112. doi: 10.1186/1752-1947-2-112 (PMC2365968; doi:10.1186/1752-1947-2-112)

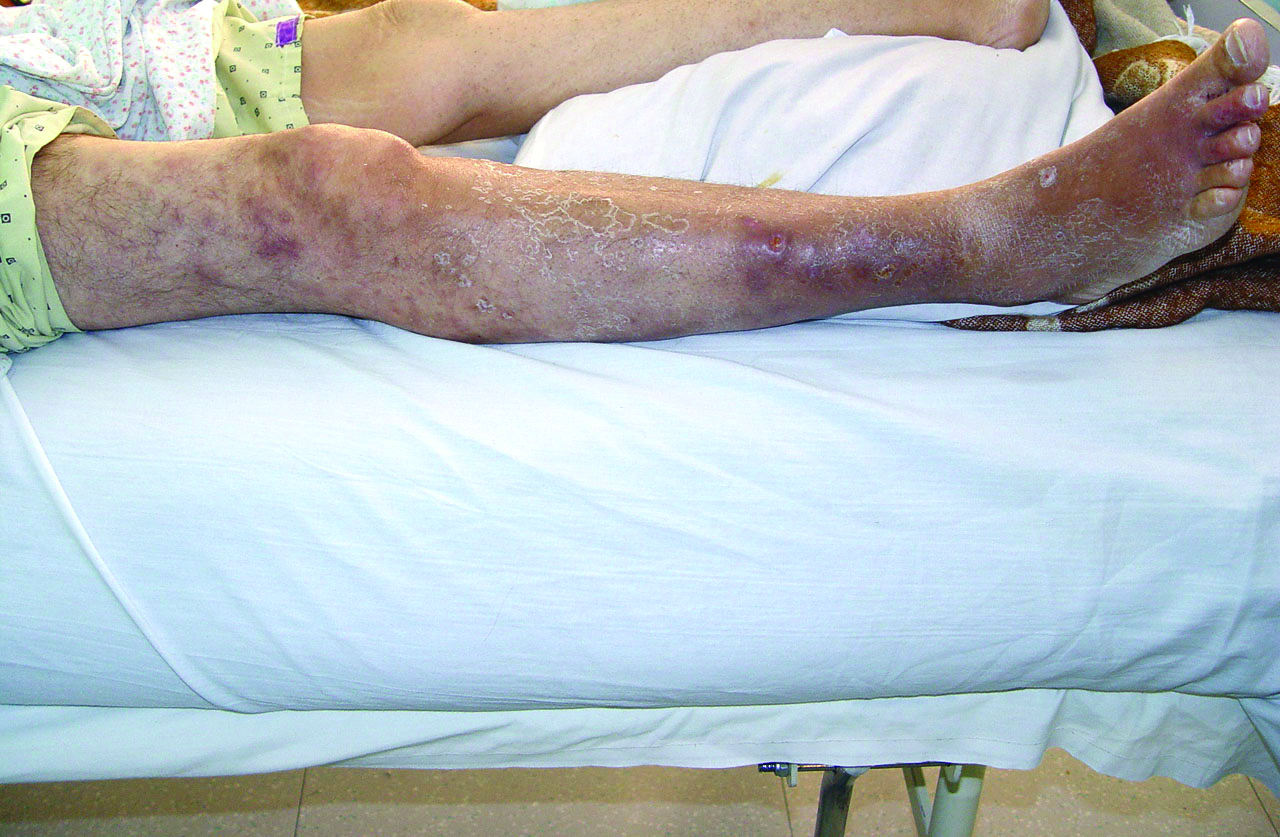

Supplement: Additional file 1 — Erythema-nodosum-like lesions of the leg and thigh [file 1752-1947-2-112-S1.tiff]

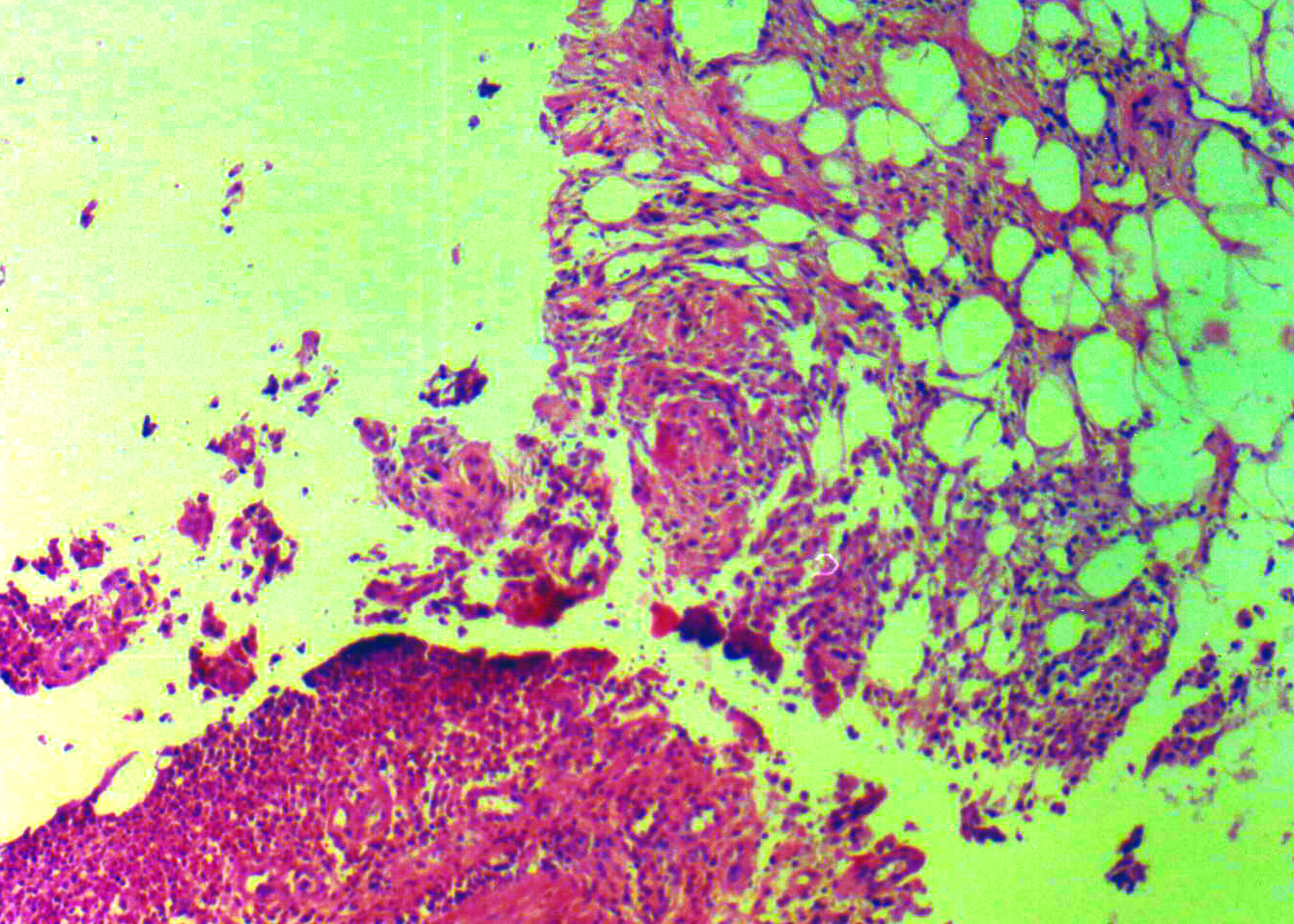

Supplement: Additional file 2 — Panniculitis [file 1752-1947-2-112-S2.tiff]

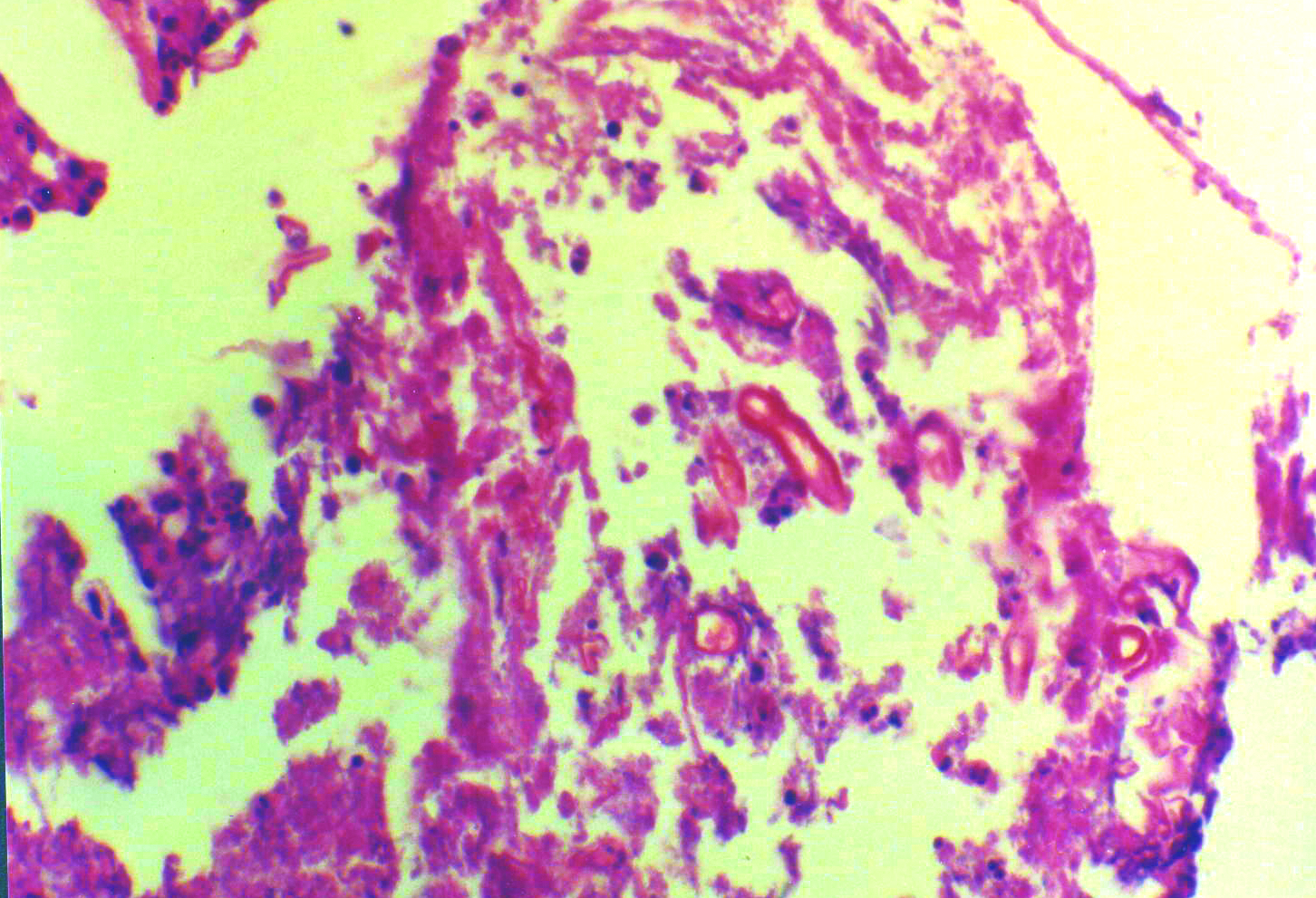

Supplement: Additional file 3 — Broad, aseptate and thin walled fungal hyphae with irregular, non-parallel contours [file 1752-1947-2-112-S3.tiff]
